# Investigation of the Factors Affecting Quality of Life in Patients with Mild to Moderate Alzheimer’s Disease in Terms of Patients and Caregivers

**DOI:** 10.3390/medicina57101067

**Published:** 2021-10-06

**Authors:** Elvan Felekoğlu, Sevgi Özalevli, Hazal Yakut, Rıdvan Aktan, Görsev Yener

**Affiliations:** 1 Graduate School of Health Sciences, Dokuz Eylül University, Izmir 35340, Turkey; 2 Department of Physiotherapy and Rehabilitation, Faculty of Health Sciences, Izmir Katip Çelebi University, Izmir 35620, Turkey; 3 School of Physical Therapy and Rehabilitation, Dokuz Eylül University, Izmir 35340, Turkey; sevgi.ozalevli@gmail.com; 4 Department of Physiotherapy, Faculty of Health Sciences, Eskişehir Osmangazi University, Eskişehir 26480, Turkey; fzthazalyakut@outlook.com; 5 Department of Physiotherapy, Izmir University of Economics, Izmir 35330, Turkey; ridvanaktan@gmail.com; 6 Faculty of Medicine, Izmir University of Economics, Izmir 35330, Turkey; gorsev.yener@ieu.edu.tr; 7 Department of Neurology, Faculty of Medicine, Dokuz Eylul University, Izmir 35340, Turkey

**Keywords:** Alzheimer’s disease, caregiver, health-related quality of life, activities of daily living, depression, sleepiness

## Abstract

*Background and Objectives*: As with other chronic diseases with limited medical treatment, the most important goal of Alzheimer’s disease (AD) treatment is to provide a better quality of life (QoL). The purpose of this study was to investigate the factors affecting the QoL of patients with mild to moderate AD in terms of patients and caregivers. *Materials and Methods*: Seventy-three home-dwelling patients with AD and their caregivers participated in this prospective, cross-sectional study. The patients were asked about their cognition, depression and a self-rating part of a QoL questionnaire. The caregivers were asked about their patients’ sociodemographic information, sleepiness, activities of daily living and a proxy rating part of a QoL questionnaire. *Results*: The self-rated QoL was higher than that provided by the proxy rating. Cognition (*p* = 0.02), sleepiness (*p* < 0.01) and depression (*p* = 0.03) were correlated with the self-rated QoL, while the patient’s independence level in activities of daily living was correlated with the proxy-rated QoL (*p* < 0.05). In regard to predicting QoL according to linear regression analysis, the following were statistically significant: depression was for total score, depression and cognition were for the self-rating and instrumental activities of daily living was for the proxy rating (*p* < 0.01). *Conclusions*: While individual factors such as psychology are an important determinant of QoL for patients with AD, objective conditions such as the independence of the patient in daily life are important for the caregiver. While evaluating the quality of life of AD patients, it is important to remember that patients and caregivers have different priorities, and the priorities of both should be taken into account when planning a treatment program.

## 1. Introduction

The incidence of dementia has increased with the aging of the world population [1]. Due to dementia, progressive damage occurs in patients’ memory, thinking, behavior, executive functions and daily life activities [1,2]. Because of these problems, dementia patients become dependent on someone else, mostly a relative, and need help in order to perform daily life activities [2].

Dementia is one of the most important diseases of our age; it is a burden on the health systems of countries, as well as on patients and their families [1]. Alzheimer’s Disease (AD), which accounts for 50–70% of all dementia cases, is the most common form of dementia. There is no cure for AD yet, and the main goal of its treatment is to ensure the optimization of the quality of life of patients [3].

According to the World Health Organization (WHO), quality of life (QoL) is an individual’s perception of their position in life in the context of the culture and value systems in which they live, and in relation to their goals, expectations, standards and concerns [4]. Improving the quality of life of those with AD is important for patients, caregivers and health care professions [4].

Quality of life is an important assessment due to its multidimensional construct, and it guides healthcare professionals in patient-centered treatment [5,6]. Due to its multidimensionality and culture specificity, QoL data may vary in different populations. “Adding life to years rather than years to life”, as a philosophy of healthy aging, emphasizes the importance of quality of life. It is not possible to reach a standard result for QoL [5]. It is inevitable that QoL and its associated factors will be evaluated differently in different populations even though the disease is the same.

Evaluation of the QoL of people with AD (PwAD) is a controversial issue, as QoL is an individual’s perception. While some studies suggest that the patient’s quality of life should be evaluated by their relative or primary caregiver, some suggest that it is a subjective datum and the patient should express it themselves [4,6,7,8,9]. Studies show that patients with mild to moderate AD (with a mini-mental state assessment of 10 points or more) can provide accurate information concerning their quality of life [10,11].

Based on the aforementioned situations, the purpose of this study is to investigate and interpret the factors affecting the QoL of PwAD in terms of patients and caregivers.

## 2. Materials and Methods

### 2.1. Participants

In this prospective, cross-sectional study, nonprobability convenience sampling was used for sample selection. Seventy-three PwAD who were diagnosed with mild to moderate AD according to the National Institute of Neurological and Communicative Disorders and Stroke and the Alzheimer’s Disease and Related Disorders Association criteria and their caregivers were recruited in this study.

The answers of the patients whose mini-mental state examination (MMSE) scores are 10 and higher are more reliable. Based on this situation, the inclusion criteria of the patients were determined as follows: getting at least 10 points in the MMSE, having mild to moderate AD and having stability in clinical situations in terms of standard medical treatment in the last 3 months. The exclusion criteria of the patients were: getting diagnosed with other types of dementia or other neurological diseases, having a comorbid disease (cardiopulmonary disease, orthopedic disease, etc.) that will may create caregiver burden, having behavioral problems (yelling, wandering, being aggressive, etc.) and having communication problems (such as seeing or hearing). The inclusion criterion of the caregiver was living with the patient and the exclusion criterion was having communication problems such as visual, hearing or speech impairments.

As the sample size was at least 10 times the number of variables, a post hoc power analysis was performed when 73 cases were reached. The power of the study was evaluated using G*Power 3.1. It was found to be 0.99 for the quality of life in Alzheimer’s disease—self-rating (a sample size of 73, an effect size of 0.5 and an alpha value of 0.05 were considered) and was found to be 0.96 for the quality of life in Alzheimer’s disease—proxy rating (a sample size of 73, an effect size of 0.16 and an alpha value of 0.05 were considered).

The mini-mental state examination was applied to the patients during the routine examination. A statement about the study was made to those with mild to moderate stages of AD and their caregivers. The patients’ quality of life and depression were evaluated after the consent form was signed by those who agreed to participate. The researchers read the questions for the patients. Sociodemographic information was asked to the caregivers of the patients and was written on the data-recording form. Evaluation forms were given to the caregivers for the questions that they should answer. They were able to ask researchers for information when needed.

### 2.2. Questionnaires

The sociodemographic information (such as gender, age, disease duration, having another disease) of PwAD was obtained from both the patients and their caregivers. The other assessment parameters were:

#### 2.2.1. Mini-Mental State Examination (MMSE)

As a set of 30 questions MMSE, is used to assess cognitive function (indicates orientation, learning, short-term memory, language use, comprehension, and basic motor skills). It ranges from 0 to 30 and low score means high cognitive impairment [12].

#### 2.2.2. Quality of Life in Alzheimer’s Disease (QoL-AD) 

This scale consists of two parts: a quality of life in Alzheimer’s disease—self-rating (QoL-AD-SR) part filled out by patients and a quality of life in Alzheimer’s disease—proxy rating (QoL-AD-PR) part filled out by caregivers. It contains 13 domains (physical health, energy, mood, living situation, memory, family, marriage, friends, self as a whole, ability to do chores, ability to do things for fun, money and life as a whole) which are rated from poor (1) to excellent (4) and provide a total score between 13 and 52. A higher score means better QoL. It can be completed by patients with mild to moderate AD [13].

#### 2.2.3. Activities of Daily Living (ADL)

The Barthel ADL scale (ranges from 0 to 100) was used to assess the skills of the patients required to perform basic daily life activities (such as feeding, personal toileting, bathing, dressing and undressing, getting on and off a toilet, controlling bladder, controlling bowel, moving from wheelchair to bed and returning, walking on level surface as well as ascending and descending stairs) [14]. The Lawton–Brody Instrumental Activities of Daily Living (IADL) scale (ranges from 0 to 7) was used to assess independent living skills (using the telephone, shopping, preparing food, housekeeping, doing laundry, using transportation, handling medications and handling finances) [15]. A higher score means more independence in both tests.

#### 2.2.4. Geriatric Depression Scale (GDS)

The short form of the GDS was used to assess the depression of the patients. It comprises 15 questions which are answered as yes or no. In this scale, 5 questions are positive, while the others are negative. In the evaluation of the scale, no answers to positive questions and yes to negative questions are matched with 1 point. A lower score means better mood [16].

#### 2.2.5. Epworth Sleepiness Scale (ESS)

The ESS is a qualitative and quantitative measurement of sleep to detect excessive daytime sleepiness. Eight questions were scored as 0, 1, 2 and 3 according to the probability of sleeping, and the total score was calculated. A high score indicates sleepiness. If the total score is greater than 10, it indicates the presence of pathological sleepiness [17].

### 2.3. Procedures

This prospective, cross-sectional study was performed in Izmir, a city in the western part of Turkey. The answering of questionnaires was done at an outpatient dementia clinic and took nearly 30 min.

Approval of the study was obtained from the ethical committee of the Ethics Committee of the Dokuz Eylül University Noninvasive Research Ethics Board (approval number: 2017/01–27, approval date: 12 January 2017). After participants and their caregivers agreed to participate in the study they signed the written permission form.

Caregivers completed the EDSS, ADL and QoL-AD-PR questionnaires related to their patients by themselves. PwAD answered the questions about their depression and QoL-AD-SR verbally.

### 2.4. Statistical Analyses

All statistical analyses were performed in the Statistical Package for the Social Sciences, version 23.0 for Windows. The Shapiro–Wilk test was used for analyzing the normal distribution of variables. If the variables were parametric, they were described by their mean and standard deviation (SD), and if they were nonparametric, they were described by their median and interquartile range.

Correlational analyses (Pearson’s r) were performed to clarify the relationships between study variables (age, disease duration, cognition, depression, sleepiness and activities of daily living) and QoL.

Stepwise backward multiple linear regression analysis was used to identify independent predictors of QoL. The model fit was assessed using appropriate residual and goodness-of-fit statistics. Statistical significance was assigned at the *p* < 0.05 level.

## 3. Results

A total of 73 home-dwelling patients and their caregivers were included in the study. In Table 1, the sociodemographic and clinical characteristics of the patients were given.

Out of the 73 patients, 46 (63%) were female. While 46 (63%) of all patients had mild-stage AD, 27 (37%) of them had moderate-stage AD. The mean age of the participants was 68.7 years (SD = 8.53) and the disease duration was 2.06 years (SD = 1.49). The mean MMSE was 19.38 ± 4.53. Patients’ self-rated quality of life scores (38.01 ± 4.12) were higher than those rated by their caregivers (31.62 ± 5.20) (Table 1).

The mini-mental state examination (r = −0.250, *p* = 0.017), GDS (r = −0.440, *p* < 0.01) and ESS (r = −0.232, *p* = 0.024) were negatively correlated with self-rated QoL, while proxy-rated QoL (r = 0.266, *p* = 0.011) was positively correlated. Proxy-rated QoL was positively correlated with ADL (r = 0.195, *p* = 0.049), IADL (r = 0.284, *p* < 0.01) and self-rated QoL (r = 0.266, *p* = 0.011). The GDS was the only metric correlated with QoL-AD total score (r = −0.383, *p* < 0.01). The correlations are shown in Table 2.

In explaining the variance in QoL, it was revealed that MMSE, GDS and proxy-rated QoL explained 30% of the variance in self-rated QoL, whereas IADL and self-rated QoL explained 12% of the variance in proxy-rated QoL. When examined in terms of QoL-AD, GDS explained 13% of it (Table 3). MMSE and GDS were identified as negatively influencing predictors of self-rated QoL, while proxy-rated QoL was identified as an independent positive predictor of better self-rated QoL. Self-rated QoL and IADL were identified as positively influencing predictors of better proxy-rated QoL. GDS was identified as a negative predictor of QoL-AD.

## 4. Discussion

In this study, the patients rated their QoL significantly higher than their caregivers, which is in accordance with the literature. Cognition, sleepiness and depression were associated with self-rated QoL, while basic and instrumental activities of daily living were associated with proxy-rated QoL.

People with Alzheimer’ disease described a better quality of life as their cognitive level decreased. Poor quality of life was found to be associated with daytime sleepiness and depressive symptoms. In terms of the caregivers, a more independent life means a better quality of life for PwAD. If the total score of the quality-of-life scale was taken into account, it would be seen that only the patient’s depressive symptoms negatively affected his quality of life, and no relationship with other symptoms would be seen. For these reasons, both the reports of patients and proxies should be considered in the treatments of PwAD.

According to the results of our study, self- and proxy-rated QoL are not related to age and disease duration. In previous studies, it is seen that the effect of age and duration of illness is a controversial issue in regard to the QoL of PwAD [7,8,10,11,18]. Similar to our study, Dewitte et al. [11] showed there is no relationship between age and quality of life but, in contrast to our results, Andrieu et al. [10] and Hongisto et al. [18] have shown that proxy-rated QoL is affected by the disease duration. According to these 2- and 5-year follow-up studies, the quality of life reported by the patient’s caregiver has significantly decreased due to disease progression, functional disorders and behavioral disorders. On the other hand, PwAD did not change their reported quality of life during the disease process as they developed mechanisms to cope with the new difficulties they encountered. Decreasing patient awareness ensures that quality of life is not affected by the disease duration [8].

Our results show that caregivers assumed that patients’ QoL worsens with impaired cognition, but this relationship was not statistically significant. When examined from the perspective of the patient, it is seen that the quality of life increases with cognitive impairments, supporting the results found in the literature [9,19]. In the study by Stites et al. [9], PwAD who were aware of their diagnosis and the disease reported more stress and depression, as well as a poorer quality of life. Quality of life does not show a linear relationship with the disease. In a study comparing mild cognitive impairment (MCI), mild AD and healthy individuals, MCI patients reported a worse quality of life than both individuals with AD and healthy individuals [19]. According to this study of Stites et al., in the MCI group awareness was high because their cognitive loss was lower, and therefore the perception of their quality of life was poor. In our study, the negative correlation between quality of life and MMSE supports this result. As a health policy, early diagnosis is important, but when we consider our results, how to inform the patient with early diagnosis is also an important situation. Information should be given about psychological changes, in addition to the physical changes that may be seen in patients. Thus, in the earlier stages when there is awareness, there may be a milder situation.

Depression and sleepiness, which were consistent with previous research, were also correlated with and contributed to lower self-rated QoL [5,20,21]. Depression, which is perceived and evaluated personally, is an important risk factor that also affects quality of life, decreasing the brain’s ability to cope with the disease. As stated in previous studies, patients with severed AD rejected depressive symptoms, while mild to moderate patients showed psychological distress. For these reasons, depression should be considered as a treatable condition from the early stages when awareness is higher, and should be treated with the most appropriate approaches for the patient.

Similar to our study, Barbe et al. measured the quality of life with OoL-AD (three scores; patient, caregiver and overall) and examined the association between depression and each of the 13 items of the QoL-AD [22]. In both the patient reports and the caregiver reports, the presence of depression influenced the three items of QoL, which consist of mood, ability to do things for fun and life as a whole. Instead of an extra depression evaluation, using these quality-of-life items may provide convenience. Studies are needed for this aim.

Another result of our study is that there is an association between a high level of daytime sleepiness and a worse quality of life. Sleep disorders and abnormal circadian rhythms are important problems which are seen in 25–60% of PwAD [5,21,23]. According to Petrovsky et al.’s state-of-the-art review, although sleep disorders are common, there is no study that summarizes how they affect QoL [21]. It also causes stress for the caregiver, due to it causing other health-related problems in the patients (depression, heart disease, decrease in functional capacity, etc.). As can be seen from our results, a patient’s socialization is reduced due to excessive daytime sleepiness, and accordingly their own quality of life perception decreases. Sleep should be considered as a treatable factor affecting quality of life and take a part in the routine evaluation of PwAD.

Activities of daily living are one of the important issues investigated in the dementia group [11,23,24,25]. In our study, according to the statements of caregivers of PwAD, better quality of life of patients is related to their independence in activities of daily living.

Subjective cognitive decline (SCD) is an important stage in which cognitive complaints occur before AD diagnosis. Since psychological conditions such as depression and anxiety also cause SCD, it is difficult to distinguish them from AD at this stage. Roehr et al. [25] found that if SCD-diagnosed patients had had disabilities in IADL, they were likely to be diagnosed with AD in the future. Due to the awareness of the caregivers, IADL is an important point that should not be neglected during the disease process.

Executive functions and ADLs are given more important by caregivers [11]. According to the results of the Barbe et al.’s study, being able to move and use the telephone create self-esteem and provide a better perceived quality of life [23]. Therefore, it is important to create a rehabilitation program for an activity related to a patient’s quality of life.

There are some limitations that should be considered while interpreting this study’s results. Non-inclusion of patients with severe AD is one of the limitations of this study. The rate of PwAD staying in nursing homes has also increased due to the increase in the disease’s incidence and care needs. Therefore, another limitation of this study was the participation of PwAD that only lived in the community. Considering these limitations, it is recommended to plan more comprehensive studies.

## 5. Conclusions

The most important goal of Alzheimer’s disease treatment is to provide a better quality of life. Because caregivers have priorities different to those of patients, both the evaluations of PwAD and the caregivers should be applied while evaluating QoL. Needs and wishes of both the patient and his proxy should be taken into account when planning the treatment program.

## Figures and Tables

**Table 1:** Sociodemographic and clinical characteristic of participants.

Patient Characteristic	Mean	SD
Age (years)	68.7	8.53
Disease duration (years)	2.06	1.49
MMSE	19.38	4.53
GDS	2.49	2.56
ESS	5.03	4.83
ADL	97.4	5.78
IADL	5.44	1.98
QOL-AD-SR	38.01	4.12
QOL-AD-PR	31.62	5.20
QOL-AD	35.88	3.61

SD, standard deviation; CDR, Clinical Dementia Rating; MMSE, mini-mental state examination; GDS, Geriatric Depression Scale; ESS, Epworth Sleepiness Scale; ADL, activities of daily living; IADL, instrumental activities of daily living; QOL-AD-SR, quality of life in Alzheimer’s disease—self-rated; QOL-AD-PR, quality of life in Alzheimer’s disease—proxy-rated; and QoL-AD, quality of life in Alzheimer’s Disease.

**Table 2:** Correlations of quality of life in Alzheimer’s disease, quality of life in Alzheimer’s disease—self-rated and quality of life in Alzheimer’s disease—proxy-rated with study variables.

Variable	QOL-AD-SR		QOL-AD-PR		QOL-AD	
	r	*p*	r	*p*	r	*p*
Age	−0.02	0.432	0.061	0.305	0.014	0.455
Disease duration	−0.045	0.351	0.136	0.126	0.031	0.399
MMSE	−0.250	0.017 *	0.144	0.112	−0.120	0.155
GDS	−0.440	<0.001 *	−0.101	0.199	−0.383	<0.001 *
ESS	−0.232	0.024 *	−0.017	0.442	−0.184	0.059
ADL	−0.185	0.058	0.195	0.049 *	−0.047	0.346
IADL	0.071	0.276	0.284	0.007 *	0.190	0.054
QOL-AD-SR	-	-	0.266	0.011 *	0.887	<0.001 *
QOL-AD-PR	0.266	0.011 *	-	-	0.682	<0.001 *

MMSE, mini-mental state examination; GDS, Geriatric Depression Scale; ESS, Epworth Sleepiness Scale; ADL, activities of daily living; IADL, instrumental activities of daily living; QOL-AD-SR, quality of life in Alzheimer’s disease—self-rated; QOL-AD-PR, quality of life in Alzheimer’s disease—proxy-rated; and QoL-AD, quality of life in Alzheimer’s disease. * Statistically significant difference (*p* < 0.05).

**Table 3:** Regression models of factors predicting quality of life in Alzheimer’s disease, quality of life in Alzheimer’s disease—self-rated and quality of life in Alzheimer’s disease—proxy-rated.

		Beta Coefficient	R^2^	Adjusted R^2^	*p* Value
QOL-AD-SR	MMSE	−0.274 (*p* = 0.003)	0.333	0.304	<0.001 *
	GDS	−0.681(*p* < 0.001)			
	QOL-AD-PR	0.211 (*p* = 0.009)			
QOL-AD-PR	QOL-AD-SR	0.313 (*p* = 0.029)	0.141	0.117	0.005 *
	IADL	0.700 (*p* = 0.019)			
QOL-AD	GDS	−0.541 (*p* = 0.001)	0.146	0.134	0.001 *

MMSE, mini-mental state examination; GDS, Geriatric Depression Scale; IADL, instrumental activities of daily living; QOL-AD-SR, quality of life in Alzheimer’s disease—self-rated; QOL-AD-PR, quality of life in Alzheimer’s disease—proxy-rated; QoL-AD, quality of life in Alzheimer’s disease. * Statistically significant difference (*p* < 0.05).

## Data Availability

The datasets used and/or analyzed during the current study are available from the corresponding author on reasonable request by elvanfelekoglu@gmail.com.
